# Editorial: Dark and Bright Side of Social Media in Current Normal

**DOI:** 10.3389/fpsyg.2022.926855

**Published:** 2022-07-06

**Authors:** Ali Nawaz Khan, Naseer Abbas Khan, Ahsan Ali, Tahir Islam

**Affiliations:** ^1^Research Center of Hubei Micro & Small Enterprises Development, School of Economics and Management, Hubei Engineering University, Xiaogan, China; ^2^Department of Industrial Economics and Project Management, South Ural State University, Chelyabinsk, Russia; ^3^School of Economics and Management, Zhejiang Sci-Tech University, Hangzhou, China

**Keywords:** social media, performance, COVID-19, educational psychology, communication, dark and bright sides of SM

## Introduction

The growth of social media worldwide has enabled individuals and organizations to facilitate their interaction, communication, social networking, and relationships (Ali et al., 2019; Pitafi et al., 2020; Khan, 2021b). Social media has enabled individuals to create and maintain social relationships and organizations, and deliver services to internal and external customers using innovative, efficient, fast, and cost-effective business models (Khan and Khan, 2019; Kakar and Khan, 2020). The growth and usefulness of social media have encouraged fortune 500 organizations to use social media to facilitate their communications with employees and reach global customers. However, this relatively rapid growth has also caused even faster growth in negative effects, mainly due to excessive use, cyberbullying, information overload, and wrong or inaccurate information being shared by social media users (Xiongfei et al., 2020; Khan et al., 2022). These negative effects represent an important threat to social media use and the need to effectively understand both the bright and dark sides of social media is urgent (Ali et al., 2019; Bano et al., 2019; Raza et al., 2020). As the relevant technical bodies and organizations are failing to catch up with the fast-growing and changing social media technologies, there is a need to further elaborate on the dark and bright sides of social media (Khan et al., 2019; Khan, 2021a).

## Summary

This Research Topic focuses on the impact of social media on individuals, teams, and organizations in the COVID-19 setting. The call for papers was open from November 11, 2020, to November 10, 2021, during the COVID-19 pandemic. Scholars were invited to submit research papers in the field of organizational psychology to two sections of Frontiers in Psychology 1- Organizational Psychology, and 2- Personality and Social Psychology. In response to this appeal, 19 research articles were submitted, with 13 being accepted and published under this theme. The Research Topic presents studies from diverse cultural and industrial settings such as manufacturing and services. It also presents new research approaches and methodologies contributing to the theory and practice in this important emerging research domain.

Keren et al. investigated and compared “Natives and Sojourners” health-protective behavior during the pandemic. They adopted a unified view to proposing a theoretical model by adapting the Health Belief Model and Institutional Theory. Their study noted that media self-efficacy, scientific self-efficacy, perceived health risks, and the perceived benefits of being protected have positive and significant effects on the definition of health-protective behavioral intentions among natives and sojourners in mainland China. Media self-efficacy, and scientific self-efficacy can play a strategic role in formulating public health-protective behavior. They recommend effective communication with sojourners during a crisis and for them to be a part of the national crisis management plan (i.e., infectious disease).

Zheng C. et al. suggested that the COVID-19 pandemic has encouraged organizations to adopt social media and digital technologies to facilitate their employees to perform routine job assignments. They further noted that COVID-19 has created challenges and uncertainties that create a problematic leader and follower relationship. Their study investigated the mediating role of psychological empowerment between the indirect relationship of unethical leadership and extra-role behavior. They further propose the moderating role of perceived organizational support in the relationship between unethical leadership and psychological empowerment and the relationship between unethical leadership and extra-role behavior. Their study focused on how the leader-follower relationship is affected in the IT industry during the COVID-19 pandemic. They show that perceived organizational support is important in mitigating the negative effects of unethical leadership behavior on the extra-role behavior of followers.

Another study by Gao et al. suggested that the useful potential of social media motivated organizations to structure their work design in a way that enables employees to benefit from social media adoption. Their study examined how social media (enterprise and public) adoption by organizations affects their designers' performance by facilitating novelty-focused product design, and efficiency-focused product design. Their empirical findings show that social media (whether for enterprises or the public) is a valuable technological tool that inspires and facilitates designers to create products that are novel and efficient. Their findings show that leaders are more satisfied with designers who produce efficiency-focused products than those who produce novelty-focused products.

In another study, Li et al. focused on how information and communication technology influence the performance of organizations during the current COVID-19 pandemic. Their study is based on complementarity theory, investigating the mediating role of employees' organizational commitment, growth mindset, and entrepreneurial orientation in the link between information and communication technology adoption and organizational performance. The empirical findings of their study show a positive mediating effect on employees' organizational commitment and growth mindset. In addition, the empirical findings do not support the mediating role of entrepreneurial orientation in linking information and communication technology with organizational performance. Their study contributes to our knowledge of the mechanisms that link information and communication technology with organizational performance during the COVID-19 pandemic.

The devastating COVID-19 pandemic has shifted the activities of employees, businesses, and students from physical to virtual work environments. The pandemic has lead to increased reliance on the internet, as people need to use social media for their work, news updates, and to post their own updates (Lindinger-Sternart et al., 2020; Zheng F. et al.), a completely different experience of social media. Several studies on the impact of social media on work productivity, performance, turnover intention, and job satisfaction have been presented since the outbreak of COVID-19 (Zivnuska et al., 2019; Cao et al., 2020; Obrenovic et al., 2020). Furthermore, various studies have examined the impact of excessive social media use on people's mental health during COVID-19 (Yu et al., 2018; Khan et al., 2021).

## Conclusion

This Research Topic brings together articles that confirm the complexity surrounding social media adoption by individuals and organizations. These articles confirm that social media has important implications for people, customers, organizations and their employees, the general public, governments, and technology and social media providers. Social media has enabled users to connect, communicate, exchange, reach customers, conduct online courses, and work from home during the COVID-19 pandemic (Ali et al., 2020; Khan, 2021; Xiongfei et al., 2021). Moreover, this increased use also brings unintended ongoing threats to the wellbeing of individuals and societies. Studies have recommended the adoption of strategic social media planning in organizations so that they can receive perceived benefits while avoiding certain dark consequences. For individuals, studies have recommended that behavioral modeling and user training are important sources for effectively using social media. However, it is important to note that research on social media is still nascent and researchers must develop comprehensive models to offer better practices for individual and organizational users.

This Research Topic enables management, social media, and psychology researchers to explore the impact of social media on the mental health, productivity, and performance of workers, professionals, business people, and students all over the world (Obrenovic et al., 2020; Zheng et al., 2020; Zhao and Zhou, 2021). During COVID-19, various scholars have observed positive aspects of social media use, particularly in developing more sophisticated and user-friendly applications for working from home and improved services in e-banking, e-learning, and e-commerce (Zheng et al., 2020; Khan, 2021; Ali et al., 2022). On the other hand, some scholars have pointed out that excessive use of social media has caused information overload (Liu et al., 2021), technostress (Nimrod, 2022), and fake news bombardment during COVID-19 (Pennycook et al., 2020), negatively impacting people's mental health (Zhao and Zhou, 2021). Similarly, findings on this theme have presented scholars with suggestions for investigating the impact of social media on people's behavior and performance that could influence future research. For example, further studies could compare the effectiveness of social media before and after COVID-19. Similarly, future studies might look into the influence of social media on the success of COVID-19 vaccine campaigns.

## Author Contributions

The Editorial was drafted by AK and AA and further edited by NK and TI. All authors contributed to the article and approved the submitted version.

## Conflict of Interest

The authors declare that the research was conducted in the absence of any commercial or financial relationships that could be construed as a potential conflict of interest.

## Publisher's Note

All claims expressed in this article are solely those of the authors and do not necessarily represent those of their affiliated organizations, or those of the publisher, the editors and the reviewers. Any product that may be evaluated in this article, or claim that may be made by its manufacturer, is not guaranteed or endorsed by the publisher.
